# A phase 2 study of GVAX colon vaccine with cyclophosphamide and pembrolizumab in patients with mismatch repair proficient advanced colorectal cancer

**DOI:** 10.1002/cam4.2763

**Published:** 2019-12-26

**Authors:** Mark Yarchoan, Chiung‐Yu Huang, Qingfeng Zhu, Anna K. Ferguson, Jennifer N. Durham, Robert A. Anders, Elizabeth D. Thompson, Noah S. Rozich, Dwayne L. Thomas, Julie M. Nauroth, Christina Rodriguez, Arsen Osipov, Ana De Jesus‐Acosta, Dung T. Le, Adrian G. Murphy, Daniel Laheru, Ross C. Donehower, Elizabeth M. Jaffee, Lei Zheng, Nilofer S. Azad

**Affiliations:** ^1^ Bloomberg–Kimmel Institute for Cancer Immunotherapy Johns Hopkins University School of Medicine Baltimore MD USA

**Keywords:** checkpoint inhibitor, colorectal cancer, immunotherapy, PD-1, vaccine

## Abstract

**Background:**

Mismatch repair proficient (MMRp) colorectal cancer (CRC) has been refractory to single‐agent programmed cell death protein 1 (PD1) inhibitor therapy. Colon GVAX is an allogeneic, whole‐cell, granulocyte‐macrophage colony‐stimulating factor ‐secreting cellular immunotherapy that induces T‐cell immunity against tumor‐associated antigens and has previously been studied in combination with low‐dose cyclophosphamide (Cy) to inhibit regulatory T cells.

**Methods:**

We conducted a single‐arm study of GVAX/Cy in combination with the PD1 inhibitor pembrolizumab in patients with advanced MMRp CRC. Patients received pembrolizumab plus Cy on day 1, GVAX on day 2, of a 21‐day cycle. The primary endpoint was the objective response rate by Response Evaluation Criteria in Solid Tumors (RECIST) version 1.1. Secondary objectives included safety, overall survival, progression‐free survival, changes in carcinoembryonic antigen (CEA) levels, and immune‐related correlates.

**Results:**

Seventeen patients were enrolled. There were no objective responses, and the disease control rate was 18% by RECIST 1.1. The median progression‐free survival was 82 days (95% confidence interval [CI], 48‐97 days) and the median overall survival was 213 days (95% CI 179‐441 days). Biochemical responses (≥30% decline in CEA) were observed in 7/17 (41%) of patients. Grade ≥ 3 treatment‐related adverse events were observed in two patients (hemolytic anemia and corneal transplant rejection). Paired pre‐ and on‐treatment biopsy specimens showed increases in programmed death‐ligand 1 expression and tumor necrosis in a subset of patients.

**Conclusions:**

GVAX/Cy plus pembrolizumab failed to meet its primary objective in MMRp CRC. Biochemical responses were observed in a subset of patients and have not previously been observed with pembrolizumab monotherapy in MMRp CRC, indicating that GVAX may modulate the antitumor immune response.

## Graphical abstract

Mismatch repair proficient colorectal cancer has been refractory to single‐agent programmed cell death protein 1 inhibitor therapy. Colon GVAX is an allogeneic, whole‐cell, granulocyte‐macrophage colony‐stimulating factor‐secreting cellular immunotherapy that induces T‐cell immunity against tumor‐associated antigens and has previously been studied in combination with low‐dose cyclophosphamide (Cy) to inhibit regulatory T cells. In this single‐arm study, we find that GVAX/Cy plus pembrolizumab results in biochemical responses but not radiographic responses.

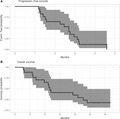

## 1 INTRODUCTION

Despite the existence of excellent screening and preventative strategies, colorectal carcinoma remains the third leading cause of death from cancer in both males and females.^1^ Cytotoxic chemotherapy remains the standard treatment for most patients with advanced metastatic colorectal cancer (CRC). Pembrolizumab, a monoclonal antibody targeting programmed cell death protein 1 (PD‐1), has demonstrated success in treating the small subset of CRC with mismatch repair deficiency (MMR‐d), also referred to as microsatellite instability (MSI).^2^, ^3^ However, little or no activity has been observed with anti‐PD1 therapy in patients with mismatch repair proficiency (MMR‐p) CRC, also referred to as microsatellite stable CRC.^2^ It is estimated that 96% of advanced CRC is the MMR‐p subtype.^3^ The development of novel therapies that can convert an immune‐checkpoint unresponsive cancer such as MMR‐p CRC into an immune checkpoint sensitive cancer remains a significant area of research.

One potential strategy for priming the tumor microenvironment (TME) for immune checkpoint therapy is to induce tumor infiltrating lymphocytes and adaptive immune resistance pathways through the use of therapeutic cancer vaccines.^4^ The GVAX colon vaccine is an allogeneic, whole‐cell, granulocyte‐macrophage colony‐stimulating factor (GM‐CSF)‐secreting cellular immunotherapy that induces T‐cell immunity against a broad range of colon cancer‐associated antigens. It consists of two CRC cell lines (SW837, SW620), and a bystander cell line transfected with a plasmid vector encoding human GM‐CSF as a vaccine adjuvant.^5^ Preclinical and clinical studies from our group and others have shown that GM‐CSF‐secreting allogeneic vaccines can increase tumor infiltrating CD8^+^ T effector cells, but these cells produce interferon gamma, leading to upregulation of the PD‐1/ programmed death‐ligand 1 (PD‐L1) pathway.^6^, ^7^ In a prior study utilizing a similar vaccine approach in pancreatic cancer, neoadjuvant treatment with pancreatic GVAX induced high levels of PD‐L1 expression on the epithelial tumor cells and led to the formation of novel vaccine‐induced, immunologically active, tertiary lymphoid aggregates; these are organized lymph node‐like structures that are not observed in tumor tissue resected from unvaccinated patients.^7^ The upregulation of immunosuppressive regulatory mechanisms by cancer vaccines suggests that cancer vaccines such as the GVAX colon vaccine may be most effective when combined with immune checkpoint inhibitors. GVAX has previously been administered with low‐dose cyclophosphamide (Cy) to deplete regulatory T cells within the tumor. An immune modulatory role of low‐dose Cy in depleting regulatory T cells and recruiting high‐avidity CD8^+^ T cells has been demonstrated in a number of pre‐clinical and clinical studies.^8^, ^9^, ^10^, ^11^, ^12^, ^13^, ^14^, ^15^

We conducted a single‐arm phase 2 study of Cy/GVAX in combination with the anti‐PD1 therapy pembrolizumab in patients with MMR‐p metastatic CRC, to test the hypothesis that Cy/GVAX, through its effects on the colon cancer TME, may modify an otherwise PD‐1 inhibitor insensitive cancer into a sensitive one. The primary objective of the trial was to determine whether the combination of Cy/GVAX plus pembrolizumab yields a clinically compelling antitumor activity. We also assessed the safety of this therapy combination, and the effect of therapy on the tumor immune microenvironment (TME) in available paired tumor biopsy specimens.

## 2 MATERIALS AND METHODS

This was an open‐label single‐arm phase 2 clinical trial conducted at the Sidney Kimmel Comprehensive Cancer Center at John Hopkins University. Patients over the age of 18 years with pathologically confirmed, MMR‐p adenocarcinoma of colorectum, who had received at least two prior lines of therapy in the metastatic setting were potentially eligible for this clinical trial. Other key eligibility criteria included the presence of Response Evaluation Criteria in Solid Tumors (RECIST) criteria 1.1 measurable disease,^16^ an Eastern Cooperative Oncology Group performance status of 0 or 1, and adequate organ function as defined by absolute neutrophil count ≥1500 cells/μL, hemoglobin >9 g/dL, platelet count ≥75 000 cells/μL, total bilirubin ≤1.5× upper limit of normal, and serum creatinine ≤2.0 mg/dL. Subjects with MSI CRC were ineligible for this study. Other key exclusion criteria included the presence of malignant small bowel obstruction within the last 6 months, clinically significant ascites, the presence of brain metastases, the presence of an active autoimmune disease that had required systemic treatment in the past 2 years, or prior treatment with a systemic immunotherapy for CRC.

### 2.1 Evaluation and treatment

**Figure 1:**
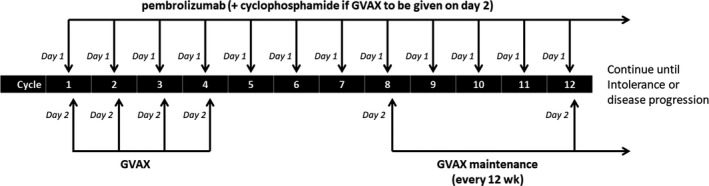
Study schema. Patients received pembrolizumab plus cyclophosphamide (Cy) on day 1, GVAX colon vaccine on day 2, of a 21 d cycle through 4 cycles, and then switched to a maintenance regimen of pembolizumab every 3 wk with Cy/GVAX given every 12 wk

The overall trial design is shown in Figure 1. Enrolled subjects initially received pembrolizumab plus Cy on day 1, the GVAX colon vaccine on day 2, of a 21‐day cycle through four cycles. After completing four cycles of therapy, subjects continued on a maintenance regimen of pembrolizumab every 3 weeks with Cy/GVAX given every 12 weeks. Cyclophosphamide was administered at 200 mg/m^2^ as an IV infusion over 30 minutes. Pembrolizumab was administered at a flat dose of 200 mg IV over 30 minutes. The GVAX colon vaccine was administered at a dose of 5 × 10^8^ colon cancer cells and 5 × 10^7^ GM‐CSF‐secreting cells given over 8‐9 intradermal injections distributed equally among the right and left thighs, and the non‐dominant arm. Lidocaine‐based topical anesthetic was applied at least 1 hour prior to vaccination at the planned vaccine sites to reduce local discomfort. The treatment protocol permitted dose delays in the setting of adverse events (AEs), but did not allow for dose reductions of any therapeutic agent.

Patients were evaluated every cycle for AEs using the National Cancer Institute Common Terminology Criteria for Adverse Events version 4.0.^17^ Imaging scans were performed at baseline and at least every 12 weeks thereafter, irrespective of the treatment schedule. Response was evaluated according to the RECIST, version 1.1^16^ as well as immune related response criteria (irRC).^18^ Upon progression of disease or discontinuation of study therapy for any other reason, patients were monitored for long‐term AEs and survival.

All patients provided written informed consent prior to enrollment, and the trial was registered under http://ClinicalTrials.gov as NCT02981524. The protocol was approved by the Institutional Review Board (IRB) at Johns Hopkins (IRB IRB00114053), and complied with the International Ethical Guidelines for Biomedical Research Involving Human Subjects and the Declaration of Helsinki.

### 2.2 Statistical methods

The study was designed to enroll up to 25 subjects, with objective response rate (ORR) by RECIST 1.1 as the primary endpoint. Enrollment was conducted in two stages so that the study could terminate early if the GVAX colon vaccine in combination with pembrolizumab was not sufficiently effective. A recently published phase 3 study showed that trifluridine/tipiracil resulted in an ORR of 1.6% (95% confidence interval [CI] 0.7%‐3%) and the agent had an overall survival benefit versus placebo in a similar patient population.^19^ Simon's minimax two‐stage design was employed to test the null hypothesis that the true ORR is 3% or less (not considered clinically compelling for this combination). A total of 15 patients were enrolled in Stage 1. If none of the 15 subjects attained an objective response, the study would be terminated. Otherwise, 10 additional subjects would be accrued in Stage 2 to target a total of 25 treated and response evaluable subjects. Three or more objective responses of 25 response evaluable subjects were pre‐established as an efficacy benchmark. This design yields 89% power at a one‐sided type I error rate of 5% when the true response rate is 20%.

Time to event data were summarized using the Kaplan‐Meier method with 95% CI derived using Greenwood's variance estimate. Pre‐ and on‐treatment anti‐carcinoembryonic antigen (CEA) antibody titers were compared using Wilcoxon's signed test, while anti‐CEA antibody titers between responders and non‐responders were compared using Wilcoxon's rank‐sum test. All reported *P* values are two‐sided; *P* values less than .05 were considered statistically significant.

### 2.3 Enzyme‐linked immunosorbent assay for anti‐CEA antibodies

A 96‐well enzyme‐linked immunosorbent assay (ELISA) plate was coated for 12 hours at 4°C with 200 ng/well of recombinant human CEACAM‐1/CD66a protein purchased from R&D Systems (Catalog #4128‐CM‐050). All following incubations were subsequently performed at room temperature, with three washes in PBS/0.05% Tween 20 (PBST) between each step. Wells were blocked with 5% milk/PBST for 2 hours. Patient sera previously collected prior to dosing at cycle 1 day 1 and prior to dosing at cycle 3 day 1 and stored at −80°C were thawed on ice and added to each well for 2 hours (1:400 dilution in 1% milk/PBST). Horseradish peroxidase‐labeled goat anti‐human antibody (1:15 000 dilution in 1% milk/PBST; Jackson Immunoresearch) was added to each well, and color development was subsequently performed with SureBlue peroxidase reagent (KPL) before reading absorbances at 450 nm. A commercial anti‐CEA antibody (MAB41281‐SP; R&D Systems) and an anti‐His tag antibody were used as positive controls. Sera from five healthy control specimens, and five uncoated wells with patient specimens known to have high‐titer anti‐CEA antibodies, were used as negative controls. All patient samples were run simultaneously, in duplicate, on a single ELISA plate.

### 2.4 Immunohistochemistry and quantitation

Manual PD‐L1 staining was performed as previously described.^20^, ^21^ A 5‐µm‐thick section was cut from one formalin‐fixed paraffin‐embedded block from each tumor specimen and mounted on glass slides. After deparaffinization and rehydration, antibody staining was performed using the SP142 clone (Spring Bioscience) and detected using the Tyramide Signal Amplification system (PerkinElmer). Slides were scanned at 20× objective equivalent (0.49 microns/pixel) using an Aperio Scanscope. A trained gastrointestinal pathologist (RAA) determined the tumor area. Quantitative image analysis (HALO; Indica Labs) was used to determine the percent of area that stained positive for PD‐L1 in tumor and its 100 μm adjacent stroma, and was reported as the percent of positive staining area. An example of annotation and analysis of PD‐L1 staining for a single subject in both pre‐ and on‐treatment biopsy serial biopsy specimens is shown in Figure S1. Necrosis area ratio was quantified based on hematoxylin and eosin staining as the percentage of necrosis area in the area of whole tumor mass.

## 3 RESULTS

### 3.1 Patient characteristics

From June through December 2017, 17 patients with advanced metastatic mismatch repair proficient (MMRp) CRC were enrolled at Johns Hopkins University and received study therapy. The original study design was to accrue to target a total of 25 treated and response evaluable subjects but included an interim analysis after the first 15 subjects were accrued. During the interim analysis, the investigators determined that the study should not continue enrollment, but due to rapid study enrollment two additional subjects had been accrued by the time that the study was closed for futility. The clinicopathological characteristics of the patients entered onto this study are shown in Table 1. The median age was 58, and the majority of patients were white, non‐Hispanic. Many patients were heavily pretreated, with most patients having received four or more prior systemic therapies for CRC.

**Table 1:** Baseline patient characteristics for all subjects enrolled on study therapy

Characteristic	n = 17
Age	Median: 58│Range: 44‐85
Sex	Male 35%│Female 65%
Race/ethnicity	White, non‐Hispanic 70.6%│Black 18%│Asian 6%
Prior systemic therapies	2, 18%│3, 29%│4, 24%│5 or above, 29%
Oxaliplatin	17, 100%
Irinotecan	17, 100%
Bevacizumab or ziv‐aflibercept	17, 100%
Cetuximab or panitumumab	6, 35%
Regorafenib	7, 41%
Trifluridine and tipiracil	8, 47%
Prior radiotherapy	11, 65%
ECOG performance status	0, 35%│1, 65%
Primary disease site	Rectum 3, 18%│Left colon 7, 41%│Right colon 7, 41%
Baseline CEA	Median: 84.8│Interquartile range: 282.5
Tumor grade	Well or moderated differentiated 13, 76%│Poorly differentiated 3, 18%│Unknown 1, 6%

Abbreviation: CEA, carcinoembryonic antigen; ECOG, eastern cooperative oncology group.

### 3.2 Efficacy

**Figure 2:**
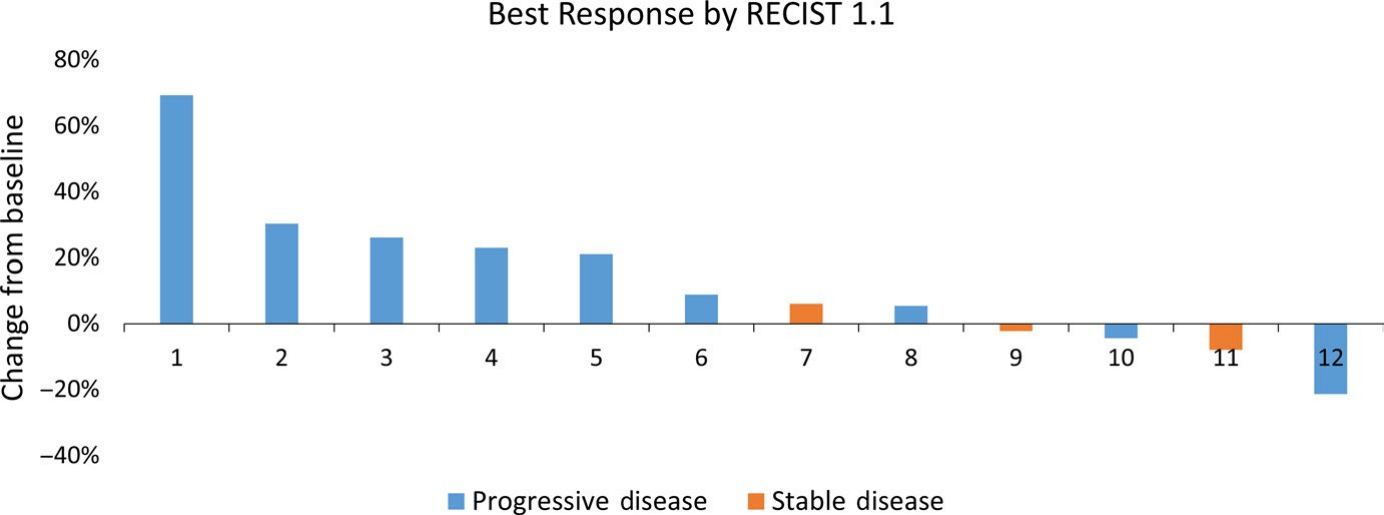
Best response by Response Evaluation Criteria in Solid Tumors (RECIST) version 1.1. among the 14 evaluable patients

Of the 17 patients enrolled on study, 14 were evaluable for objective response. The remaining three patients were withdrawn from study therapy for early clinical progression. There were no objective responses to study therapy. Response Evaluation Criteria in Solid Tumors version 1.1 responses for all evaluable subjects are shown in Figure 2. Of evaluable subjects, three had stable disease by RECIST 1.1, whereas 11 patients had progressive disease as a best response to study therapy. Three patients with PD had mixed responses to therapy, with multiple lesions shrinking but the interval appearance of new lesions. By irRC, five patients had SD and nine patients had PD as a best response to therapy. Two patients who had mixed responses to study therapy went on to have objective responses with their subsequent therapy (one receiving regorafenib and one receiving trifluridine/tipiracil).

**Figure 3:**
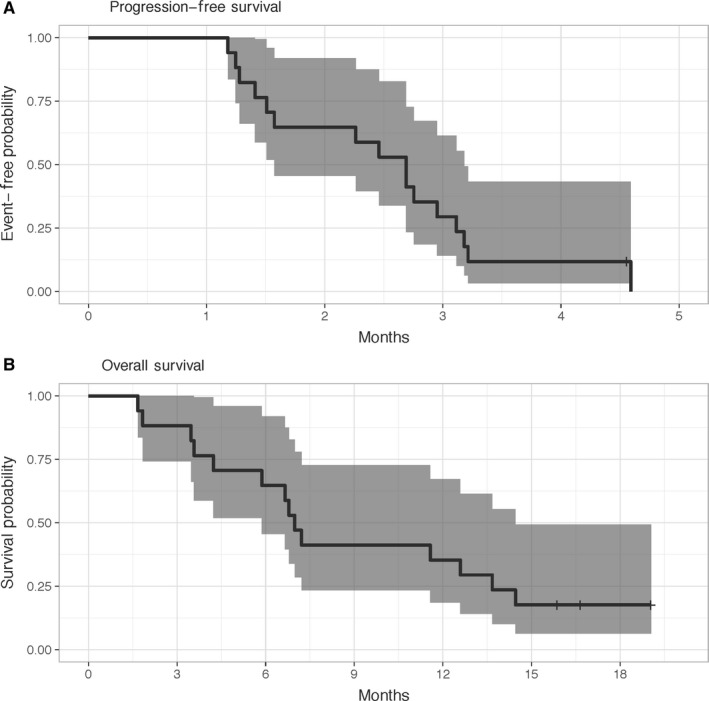
Kaplan‐Meier curves for PFS and OS with 95% confidence intervals for all 17 enrolled subjects

The overall disease control rate (DCR) on an intent‐to‐treat basis was 3 of 17 (18%) by RECIST and 5 of 17 (29%) by irRC. Figures 3A and B show the Kaplan‐Meier curves for progression‐free survival (PFS) and overall survival (OS) for all 17 study patients. The median progression‐free survival was 82 days (95% CI 48‐97 days) and the median survival was 213 days (95% CI 179‐441 days). The 6‐month OS was 65% (95% CI 46%‐92%).

**Figure 4:**
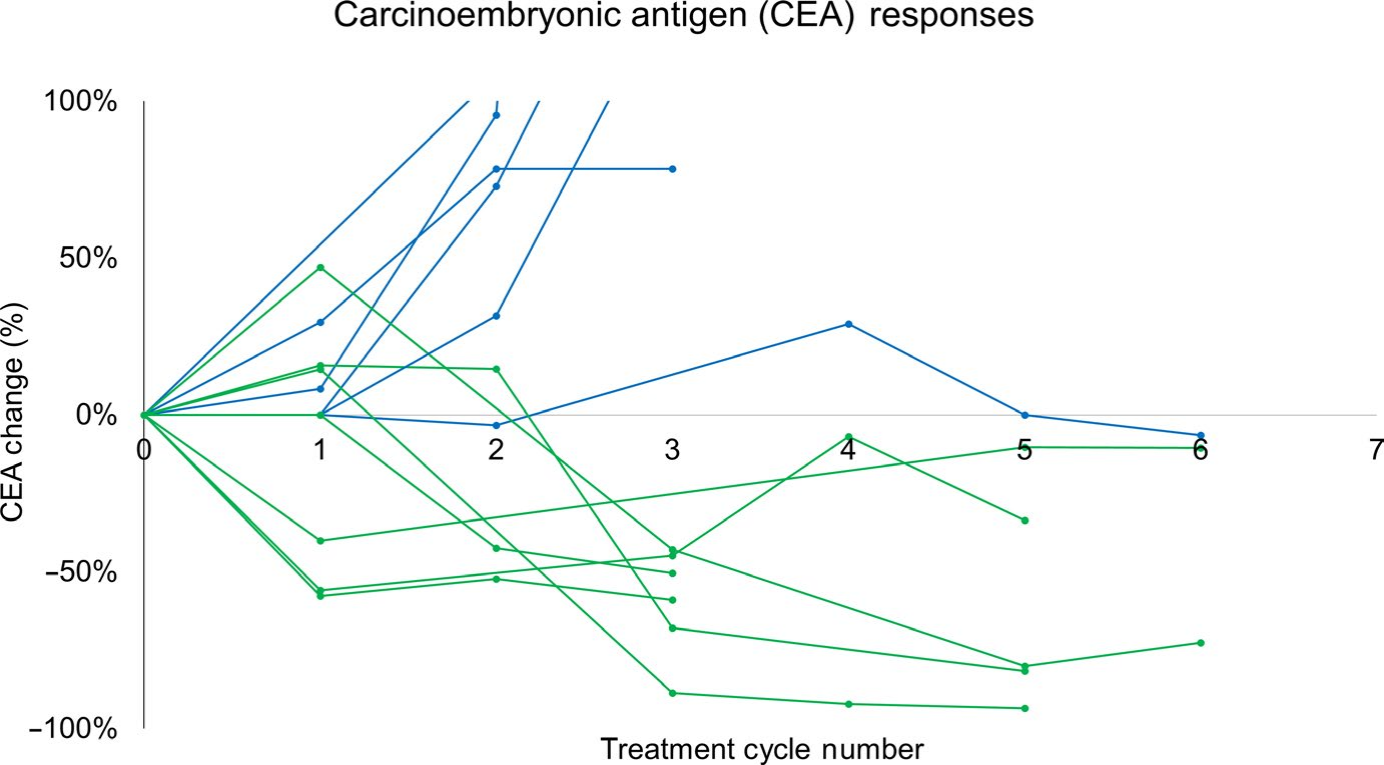
Biochemical responses to study therapy (carcinoembryonic antigen [CEA] declines of >30%) were observed in 7/17 participants (41%). Change in CEA values for all subjects are shown below over the duration of study therapy. Subjects achieving a best change from baseline in CEA measurement of 30% or more are shown in green

All 17 patients had an elevated CEA at baseline, and CEA levels were followed for the duration of study therapy for all patients. Change in CEA values for all subjects over the duration of study therapy is shown in Figure 4. A best change from baseline in CEA measurement of 30% or more was observed in 7/17 participants (41%). Among these CEA responders, CEA responses were usually observed within the first or second cycle of therapy, and persisted throughout the study period. All patients with CEA measurements available after discontinuation of study therapy had rising CEA levels shortly after study therapy was discontinued. CEA responses were uncorrelated with radiographic responses to study therapy. Of the seven patients with CEA responses, only one patient had stable disease by RECIST, whereas six patients had progressive disease as a best response to therapy.

**Figure 5:**
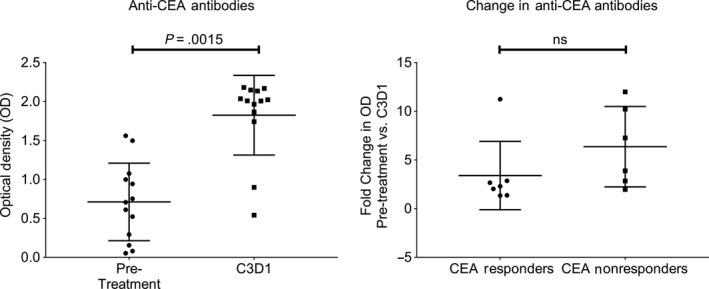
Paired analysis of pre‐ and on‐treatment sera showed that anti‐ carcinoembryonic antigen (CEA) antibody titers increased in 13/13 patients with study therapy (*P* = .0015), but the change in anti‐CEA antibodies was not correlated with CEA response (*P* = .11). *P*‐value for comparing pre and on treatment anti‐CEA antibody titers was obtained using Wilcoxon's signed test, while *P*‐value for comparing CEA responders and non‐responders was obtained using Wilcoxon's rank sum test

To investigate why marked biochemical responses to study therapy were observed with study therapy and were uncorrelated with radiographic responses to study therapy, we measured patient antibodies against CEA from stored clinical trial serum samples using recombinant CEA in an ELISA. We hypothesized that patients may have made antibodies against CEA that interfered with the measurement of the tumor marker. Among the 13 patients with paired pre‐treatment serum and treatment cycle 3 day 1 serum available for analysis, all patients had increases in anti‐CEA antibodies, and this change was significant (median pre‐ and on‐treatment anti‐CEA antibodies: 0.71 and 2; median fold increase 2.8; *P* = .002). However, the change in anti‐CEA antibody titers was not significantly different among the CEA responders and non‐responders (median anti‐CEA antibodies in responders and non‐responders, 2.3 and 5.6; *P* = .11) (Figure 5).

### 3.3 Adverse events

The median duration of treatment was 12 weeks. One patient (6%) discontinued treatment because of treatment toxicity. All grade 1‐2 treatment‐related toxicities, and all grade 3/4 toxicities regardless of attribution are listed in Table 2. There were no treatment‐related deaths and the observed treatment‐related toxicities were predominantly of mild severity. Consistent with prior clinical experiences, 16 patients experienced mild GVAX colon vaccine injection site reactions such as pain, swelling, or itching at the vaccine injection site. Injection site reactions were managed effectively with topical lidocaine or aloe vera and usually resolved within a few days of vaccine treatment. Other common treatment‐related AEs were fatigue/lethargy (29% of patients), hypothyroidism (24%), flu‐like symptoms (12%), rash (12%), and pruritus (12%).

**Table 2:** All grade 1‐2 treatment‐related toxicities, and all grade 3‐4 toxicities regardless of attribution

Event	Treatment‐related grade 1‐2		Grade 3‐4 (any attribution)	
	No of patients	%	No of patients	%
Cardio‐renal				
Hyponatremia			1	6
Constitutional				
Fatigue or lethargy	5	29		
Flu‐like symptoms	2	12		
Hot flashes	1	6		
Myalgias	1	6		
Dermatologic				
Injection site reactions	16	94		
Pruritus	2	12		
Rash	2	12		
Endocrine				
Hypothyroidism	4	24		
Gastrointestinal				
Anorexia	1	6		
AST elevation			1	6
Hematologic				
Anemia (hemolytic)			1	6
Anemia (non‐hemolytic)			1	6
Other				
Corneal transplant rejection			1	6
CPK increased			1	6
Hip fracture			1	6
Pulmonary				
Shortness of breath			1	6

Abbreviation: AST, aspartate aminotransferase; CPK, creatine phosphokinase.

Two patients had severe (grade 3+) AEs that were attributed to study therapy. One patient had an episode of grade 3 hemolytic anemia with a lactic acid dehydrogenase peaking at 766 and multiple schistocytes observed on a peripheral blood smear. The patient's antiglobulin test (DAT) was weakly positive and the patient was treated with prednisone with eventual complete resolution of the anemia. The second severe AE was an episode of grade 3 bilateral corneal allograft rejection in a patient with bilateral corneal transplants that had been performed for keratoconus approximately 30 years prior. The patient presented with symptoms of acutely decreased visual acuity, photophobia, and tearing and was found to have epithelial edema and keratic precipitates on slit lamp exam. The patient was treated with systemic and intraocular steroids with stabilization of further vision changes, but the patient's vision did not resolve to baseline. Both of these serious AEs are known potential risks of pembrolizumab and were attributed to pembrolizumab and not the GVAX vaccine.

### 3.4 Analysis of PD‐L1 expression and tumor necrosis on serial biopsy specimens

**Figure 6:**
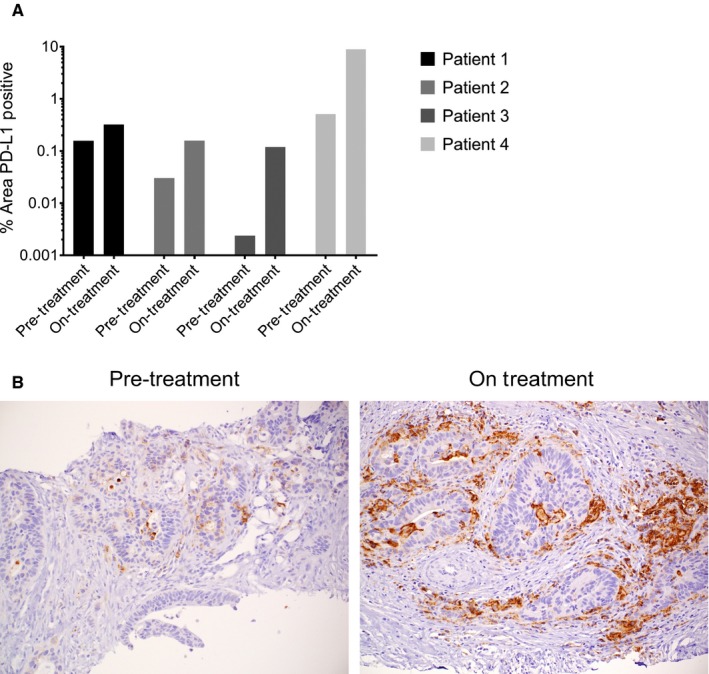
A, All four patients with serial biopsy specimens available for analysis had an increase in PD‐L1 expression at week 6 as compared to the pre‐treatment biopsy specimen. Data are displayed as the log of the percent area staining positive for PD‐L1, in the area of tumor and its 100 μm adjacent stroma. B, Pre‐ and on‐treatment PD‐L1 staining is shown for Patient 4. In this subject, PD‐L1 staining increased from 0.52% area to 8.96% area with therapy

Four patients underwent serial biopsies before treatment and after completing 6 weeks of therapy. All subjects had low levels of PD‐L1 expression at baseline (<1% tumor area). In all four subjects, there was a numeric increase in PD‐L1 expression within the tumor area and its 100 μm adjacent stroma in post‐treatment biopsies, relative to baseline (Figure 6A). However, PD‐L1 expression remained low (<1% tumor area) in 3/4 subjects even after treatment. In 1/4 subjects, PD‐L1 expression increased substantially from 0.52% area to 8.96% area with therapy (Figure 6B). In addition, three of four patients had visible tumor necrosis (>10% necrosis) in the on‐treatment biopsy specimen, and tumor necrosis was not observed in any pre‐treatment specimens.

## 4 DISCUSSION

We conducted a single‐arm clinical trial, the GVAX colon vaccine in combination with the anti‐PD1 therapy pembrolizumab in patients with MMR‐p metastatic CRC. To our knowledge, this is the first clinical trial combining an allogenic whole‐cell vaccine with an immune checkpoint inhibitor in MMR‐p metastatic CRC, and was designed to test the hypothesis that a vaccine can modify an otherwise PD‐1 inhibitor insensitive cancer into a sensitive one. Although the treatment had acceptable toxicity, none of the first 17 patients demonstrated an objective radiographic response to therapy and the trial failed to meet its primary efficacy objective.

One notable finding from this study was that multiple subjects had a significant decline in their tumor marker level with study therapy. It is unclear why biochemical responses did not translate into radiographic responses for subjects on study therapy. We initially hypothesized that the GVAX colon vaccine may have induced anti‐CEA antibodies that interfered with the laboratory measurement of CEA. In initial support of this hypothesis, we found that patients on study therapy with available paired research bloods did increase their anti‐CEA antibody titers. However, similar increases in anti‐CEA antibodies were observed among subjects with and without biochemical responses. This argues against the hypothesis that CEA responses were related to interference in the clinical laboratory test by therapy‐induced antibodies. We believe that the biochemical responses may represent some degree of tumor elimination, but may have been insufficient to manifest as a radiographic response.

While 41% of participants in this trial had a best change from baseline in CEA measurement of 30% or more, 0 of 18 patients (0%) had biochemical responses in the largest available clinical trial of pembrolizumab monotherapy in a similar population of MMR‐p metastatic CRC.^2^ The significant declines in CEA observed in this clinical trial, and the absence of such biochemical responses in prior clinical trials of pembrolizumab monotherapy, indicate that the GVAX colon vaccine in combination with Cy can modulate the immune response to a PD‐1 inhibitor. It is also notable that two patients with mixed responses with study therapy went on to have objective responses with their subsequent therapy. The therapies these two patients received after coming off study (regorafenib and trifluridine/tipiracil) are not normally associated with clinical responses, suggesting the possibility of tumor re‐sensitization to subsequent therapy or delayed responses to study therapy. An observed increase in PD‐L1 expression and tumor necrosis observed in serial biopsies from a subset of patients further support the biological activity of this treatment combination, although it is also clear that the addition of other agents will be required to reach a threshold of radiographic clinical responses in this patient population.

In conclusion, the combination of the GVAX colon vaccine and the PD‐1 inhibitor pembrolizumab failed to induce radiographic responses in patients with MMRp CRC. These results underscore the challenge of extending the benefits of anti‐PD‐1 therapies to PD‐1 inhibitor insensitive cancers, such as MMRp CRC, through novel treatment combinations that may modulate the TME. Although this treatment does not warrant further investigation in this disease population, the GVAX colon vaccine continues to show potential for modulating the TME and may warrant additional investigation in combination with other novel agents. Further evaluation of the GVAX colon vaccine in combination with other systemic immunotherapies is planned.

## ETHICS APPROVAL AND CONSENT TO PARTICIPATE

All patients provided written informed consent prior to enrollment, and the trial was registered under http://ClinicalTrials.gov as NCT02981524. The protocol was approved by the Institutional Review Board (IRB) at Johns Hopkins (IRB IRB00114053), and complied with the International Ethical Guidelines for Biomedical Research Involving Human Subjects and the Declaration of Helsinki.

## CONSENT FOR PUBLICATION

Not applicable.

## CONFLICT OF INTERESTS

Mark Yarchoan: consulting or advisory role: Eisai; Exelixis Inc; research funding: Bristol‐Myers Squibb (Inst); Exelixis Inc (Inst); Genentech/Roche (Inst); Merck (Inst). Ana De Jesus‐Acosta: consulting or advisory role: Merck. Dung T. Le: Honoraria: Merck; consulting or advisory role: Bristol‐Myers Squibb; Merck; research funding: Aduro Biotech; Bristol‐Myers Squibb; Merck. Dan Laheru: patents, royalties, other intellectual property: Colon GVAX. Elizabeth M. Jaffee: consulting or advisory role: Adaptive Biotechnologies; CStone Pharmaceuticals; Dragonfly Therapeutics; Genocea Biosciences; Incyte; research funding: Aduro Biotech; Amgen; Bristol‐Myers Squibb; Corvus Pharmaceuticals; patents, royalties, other intellectual property: I developed GVAX which is being tested in the clinics. It was licensed to Adoro Biotech. If it becomes commercial, I will have the potential to receive royalties. Also, I have received milestone payments yearly; travel, accommodations, expenses: Bristol‐Myers Squibb; Genocea Biosciences; MedImmune. Lei Zheng: stock and other ownership interests: Z and L International Medical; consulting or advisory role: AstraZeneca; Biosynergies; Merck; Merrimack; novarock; research funding: Amgen; Bristol‐Myers Squibb; Gradalis; Halozyme; ITeos Therapeutics; Merck; patents, royalties, other intellectual property: GVAX, licensed to Aduro Biotech. Nilofer Saba Azad: Honoraria: AMAG Pharmaceuticals; Bayer/Onyx; consulting or advisory role: AMAG Pharmaceuticals; DAVAOncology; research funding: Agios (Inst); Array BioPharma (Inst); Astex Pharmaceuticals (Inst); Bristol‐Myers Squibb (Inst); Celgene (Inst); Genentech (Inst); Intensity Therapeutics; Merck (Inst); Syndax (Inst).

## AUTHORS' CONTRIBUTIONS

MY, NSA, EMJ, and LZ contributed to the conception and overall design of the study. MY, CYH, and NSA wrote the study protocol. The patients were treated by MY, NSA, and AKF. All authors contributed to the acquisition, analysis, or interpretation of data. MY and NSA drafted and revised the manuscript. All authors reviewed, edited, and approved the final manuscript.

## Data Availability

The clinical data that support the findings of this study will be openly available in http://clinicaltrials.gov (https://clinicaltrials.gov/ct2/show/NCT02981524); any additional data that support the findings of this study are available from the corresponding author upon reasonable request.
