# Supplementary figures and images for: A phase 2 study of GVAX colon vaccine with cyclophosphamide and pembrolizumab in patients with mismatch repair proficient advanced colorectal cancer
Source: Cancer Med. 2019 Dec 26;9(4):1485–94. doi: 10.1002/cam4.2763 (PMC7013064; doi:10.1002/cam4.2763)

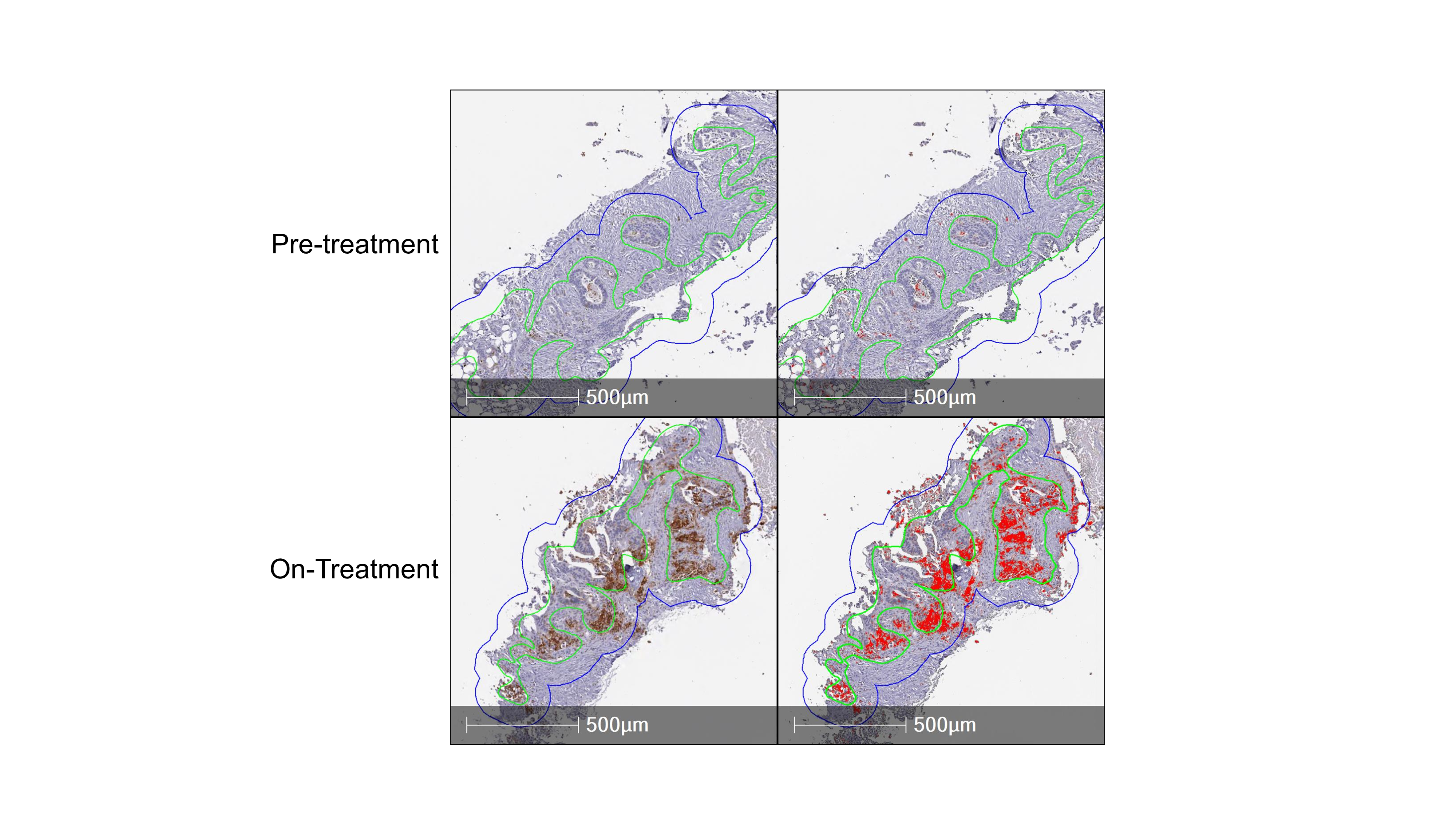

Supplement: Supplementary file 1 [file CAM4-9-1485-s001.tif]
