# Supplementary material for: A phase 2 study of GVAX colon vaccine with cyclophosphamide and pembrolizumab in patients with mismatch repair proficient advanced colorectal cancer
Source: Cancer Med. 2019 Dec 26;9(4):1485–94. doi: 10.1002/cam4.2763 (PMC7013064; doi:10.1002/cam4.2763)
Supplement: Supplementary file 2 [file CAM4-9-1485-s002.docx]

Figure S1: Example of annotation and quantitative image analysis of PD-L1 staining for a single subject in both pre- and on-treatment biopsy serial biopsy specimens. Tumor Area was annotated in green and the analyzed area was annotated in blue, which included the tumor area and its 100 μm adjacent stroma (left panels). Automated threshold detection of PD-L1 is represented with red coloring (right panels).
